# Expression of GLUT-1 and SGLT-2 in the Chorioretina of Streptozotocin-induced Diabetic Rats After Administration of Empagliflozin

**DOI:** 10.18502/jovr.v20.15231

**Published:** 2025-09-02

**Authors:** Lia Meuthia Zaini, Arief S Kartasasmita, Tjahjono D Gondhowiardjo, Maimun Syukri, Ronny Lesmana

**Affiliations:** ^1^Faculty of Medicine, Department of Medical Education, Syiah Kuala University, Banda Aceh, Indonesia; ^2^Department of Ophthalmology, Zainoel Abidin Hospital, Banda Aceh, Indonesia; ^3^Faculty of Medicine, Padjadjaran University, Bandung, Indonesia; ^4^Department of Ophthalmology, Cicendo Eye Hospital, Bandung, Indonesia; ^5^Faculty of Medicine, Department of Ophthalmology, Indonesia University, Jakarta, Indonesia; ^6^Department of Ophthalmology, Cipto Mangunkusumo Hospital, Jakarta, Indonesia; ^7^Department of Internal Medicine, Zainoel Abidin Hospital, Banda Aceh, Indonesia

**Keywords:** Chorioretina, Empagliflozin, GLUT-1, Metformin, SGLT2

## Abstract

**Purpose:**

To assess the effect of empagliflozin on the expression of SGLT-2 and GLUT-1 in the chorioretina of streptozotocin-induced diabetic rats.

**Methods:**

An *in vivo* experimental study was performed on Wistar rats. After a 2-week adaptation period, the rats were allocated to one of four groups. The group allocations were as follows: (K1) negative control (no intervention); (K2) positive control injected with streptozotocin (STZ) without treatment; (K3) positive control injected with STZ and treated with metformin; and (K4) positive control injected with STZ and treated with empagliflozin. SGLT-2 and GLUT-1 expressions were examined using the Western blot technique.

**Results:**

SGLT-2 was expressed in the chorioretinal tissue of all groups; administration of SGLT-2 inhibitors could suppress its expression. The average band thickness indicated that SGLT-2 expression was higher in diabetic rats compared to rats with normal sugar levels. GLUT-1 expression was noticed in all groups based on the chorioretinal examination. It did not change in the metformin group, whereas the empagliflozin group showed a significant decrease in the GLUT-1 expression.

**Conclusion:**

Both metformin and empagliflozin suppress the expression of SGLT-2 and GLUT-1 in the chorioretina of diabetic rats. In comparison, empagliflozin seems to be more potent in reducing this expression.

##  INTRODUCTION

Diabetic retinopathy (DR) is one of the most common microvascular complications in people with diabetes mellitus (DM). DR prevalence increases with longer duration of diabetes, reaching an overall rate of 30%.^[1,2]^ DR is also one of the leading causes of blindness worldwide, as chronic high blood glucose levels impair the anatomy and function of retinal capillaries.^[1,3]^ Adequate control of blood glucose levels is necessary to prevent DM complications and hinder DR progression.

Sodium glucose co-transporter-2 (SGLT-2) inhibitors are a new class of glucose-lowering drugs that reduce plasma glucose levels by blocking the reabsorption of glucose in the kidney.^[4]^ SGLT-2 inhibitors improve glucose control by increasing glucosuria, natriuresis, and osmotic diuresis, which leads to lower arterial blood pressure; these effects partly explain the cardioprotective attribute of these drugs.^[5]^ In addition to enhancing glucosuria, these inhibitors achieve significant weight loss by reducing dyslipidemia through altering lipid metabolism at the cellular level.^[6,7]^ As a result, improving hypertension, glucose control, and dyslipidemia management may hinder the progress of DR.^[8]^ Besides, SGLT2 supports the nutrient metabolism of the eye and has DR protective effects.^[9]^

SGLT-2 and glucose transporter-1 (GLUT-1) facilitate the movement of glucose into cells through various mechanisms.^[10,11]^ Despite the early view that the quantity of SGLT-2 in the eye was relatively small compared to other organs, SGLT-2 plays a crucial role in the development of DR. Previous studies by Wakisaka on bovine retina showed that SGLT-2 was expressed on retinal pericyte cells, the main component of the blood–retinal barrier (BRB).^[12]^ Retinal pericytes function as glucose sensors, regulating cellular tone in response to fluctuations in extracellular glucose concentration. SGLT-2 plays a vital role in glucose transport from extracellular to intracellular pericyte cells, with a glucose-to-sodium binding ratio of 1:1. Under conditions of high blood glucose, the entry of glucose into pericytes via SGLT-2 increases approximately twofold. Sodium enters the pericytes together with glucose and is pumped out of the cell by Na+–K+ ATPase to maintain cellular homeostasis. However, accumulation of sorbitol and activation of Protein Kinase C—originating from excess glucose accumulation in retinal pericytes—can inhibit the Na+–K+ ATPase pump, resulting in increased intracellular sodium and intracellular edema. This loss of pericytes causes the ratio of retinal endothelium to pericytes to drop drastically from 1:1 in normal eyes to 4:1 in eyes with DR.^[12,13]^

GLUT-1 is the major glucose transporter in the retinal pigment epithelium (RPE), which presents in the apical and basal membranes of the RPE cells as well as in the rod and cone photoreceptors. It also presents in the retinal ganglion cells and Müller cells. Glucose uptake via GLUT-1 is essential for the renewal of the photoreceptor outer segment, thereby affecting visual function and rod cell survival. Previous studies reported that GLUT-1, encoded by the *S1c2ai* gene, could impair retinal pathophysiology in diabetic model rats.^[14]^ Holoman et al found that DR was associated with increased levels of GLUT-1 in the retina, suggesting that decreased GLUT-1 might protect the retina. Additionally, GLUT-1 reduction in retinal neurons and Müller glial cells provided protection against DR, whereas GLUT-1 reduction in RPE did not. As a result, manipulating GLUT-1 levels in retinal neurons was proposed as a therapeutic target in the prevention and treatment of DR.^[14]^

Notwithstanding previous studies on SGLT-2 inhibitors to delay the progression of DR, the mechanism of these drugs is still largely unknown. The expression of SGLT-2 in renal physiology is well-documented, whereas its expression in the eye is limited. For instance, it has been established that streptozotocin (STZ) induces DNA damage and cell death in beta cells, leading to a significant reduction in insulin production.^[14]^ However, a growing number of ophthalmologists are interested in exploring the potential role of SGLT-2 inhibitors in preventing DR. As a result, this study aimed to assess the effect of empagliflozin on the expression of SGLT-2 in the chorioretina of STZ-induced diabetic rats. The reason for choosing empagliflozin in this study is that, together with dapagliflozin, it is a type of SGLT-2 inhibitor that is already available in Indonesia. In addition, this study aimed to evaluate the possible pathways of empagliflozin action, beyond its blood glucose-lowering properties, through assessing the expression of GLUT-1 as a principal glucose transport protein in the chorioretina.

##  METHODS 

### Study Design and Setting 

This *in vivo* experimental study used a posttest-only, control-group design. Wistar rats were induced with diabetes by injecting STZ, which is commonly used to induce type 1 diabetes in rat models. Preparations started in June 2020, while the samples were treated from October 2020 to January 2021. The research was conducted at the Physical Laboratory and the Central Laboratory of Padjadjaran University, Bandung, Indonesia.

### Sample Size and Randomization

The sample size was determined using the Federer formula, which is commonly used in experimental animal research. Using an appropriate formula is crucial to ensure that the sample size is sufficient for statistical validity while minimizing the use of experimental animals. This approach aligns with the 3Rs principle, specifically Reduction. Accordingly, each of the four groups comprised 6 rats (24 rats in total). Randomization was carried out using the Completely Randomized Design (CRD), whereby numbers were assigned to the test animals and draws determined the group allocation.

### Animal Model

In this animal study, 24 male Wistar rats, aged 8 to 10 weeks and weighing approximately 250 to 350 grams, were used. The rats were obtained from the BioFarma Laboratory in Bandung, belonged to the same strain, and were exposed to the same environment, consuming the same food. Animal care was implemented to ensure the welfare of experimental animals at every stage. The rats were housed in special cages equipped with unrestricted access to drinking water, appropriate ventilation, and a 12-hour light/dark cycle. Each cage contained a maximum of four rats to ensure optimal living conditions. Moisture- and odor-absorbent bedding was used, cleaned, and changed regularly, twice a week. Food was routinely given, supervised by laboratory assistants and cage keepers.

The inclusion criteria were healthy male Wistar rats, aged 8–10 weeks, with a body weight of 250–350 grams. The rats were allowed to engage in normal activities without external intervention by the researchers. The exclusion criteria were rats that were sick or died during the study, and rats in which blood glucose levels did not increase after the administration of STZ.

### Intervention

Rats that met the study criteria were fed a standard diet for adult rats and housed in a comfortable place with sawdust as a bedding medium. The research was conducted in Bandung city, where temperatures fluctuated between 20 and 28 ${}^{\circ }$ C, and hourly humidity ranges varied from 50% to 97%. After the habituation/adaptation period, the rats were induced to develop diabetes using STZ and were allocated into four groups, each consisting of six rats. Group allocation was as follows:

• Group 1: Negative control group, normal rats (without STZ) that were not given any drug;
• Group 2: Positive control group, diabetic rats (with STZ) that were not given any drug;
• Group 3: Diabetic rat group (with STZ) that was given metformin; and
• Group 4: Diabetic rats (with STZ) that were given an SGLT2 inhibitor drug (empagliflozin).

Drug administration was carried out for 8 weeks, and the drug was delivered via a probe once daily in the morning. The selected dose was determined based on previous studies that utilized metformin and empagliflozin in rats. The reason for choosing empagliflozin in this study is that it is a type of SGLT-2 inhibitor, along with dapagliflozin, which is available in Indonesia. As a result, it is considerably affordable and accessible. In addition, dose adjustment is uncomplicated due to numerous studies using empagliflozin in diabetic rat models. The dosages were 100 mg/kgBW for metformin and 30 mg/kgBW for empagliflozin. Rat models with blood glucose levels > 250 mg/dL in three examinations were included in this study. After euthanasia, enucleation was performed to obtain samples of the mouse retina, and a Western blot examination was carried out to evaluate the expression of GLUT-1 and SGLT-2. The antibodies used were obtained from Abcam: Anti-Glucose Transporter GLUT-1 antibody [SPM498] (ab40084) for GLUT-1 and anti-SGLT2 antibody (ab37296) for SGLT-2.

**Figure 1:**
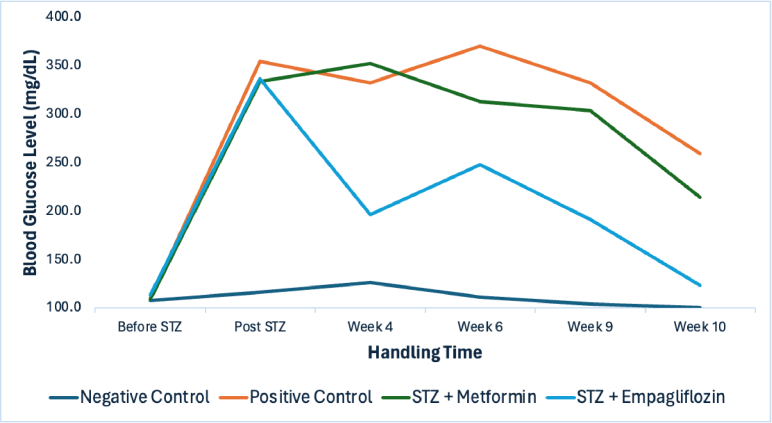
Mean of blood glucose levels of rats in each group before STZ, after injection of STZ (baseline/before therapy), at weeks 4, 6, 9, and 10 (after the administration of antidiabetics for 8 weeks). Negative Control: nondiabetic rat group. Positive Control (STZ): a group of diabetic rats without any treatment. STZ+METFORMIN: Metformin-treated diabetic rats. STZ+EMPAGLIFOZIN: Empagliflozin-treated diabetic rats.

### Western Blot Examination

The tissue preparation process began with the collection of a sample of approximately 20 mg, which was then crushed and placed into a container. Radioimmunoprecipitation (RIPA) buffer was added to prevent protein denaturation. The sample was then centrifuged at 12,000 rpm to settle, and then the lysate was extracted. Bromothymol blue dye was added to track protein movement, and the mixture was denatured at 95 ${}^{\circ }$ C for 5 minutes, followed by cooling. The precipitated samples were loaded into SDS-PAGE gel electrophoresis wells, where proteins were separated based on charge, isoelectric point, and molecular weight under an electric current.

Following electrophoresis, proteins were transferred from the gel to a nitrocellulose membrane via electroblotting. The process involved arranging a sandwich of filter paper, sponge, and membrane between the gel and a filter paper cassette, then passing an electric current to facilitate protein transfer. The gel, soaked in transfer buffer and methanol, was placed on the membrane, forming a sandwich with additional filter paper and sponge. This assembly was placed in a Mini Blot Module and inserted into a Mini Gel Tank to complete the transfer over one to two days. Blocking was then performed using casein or BSA to prevent nonspecific antibody binding to the nitrocellulose paper, followed by the addition of primary antibodies specific to the target protein.

Secondary antibodies labeled with enzymes such as alkaline phosphatase or horseradish peroxidase (HRP) were then added. These antibodies bound to the primary antibodies to form the Ag–Ab complex. The enzyme-substrate reaction produced a visible, colored product, allowing visualization of protein bands on the membrane. Western blotting served as both a qualitative and quantitative method for protein analysis. Band thickness was quantified using ImageJ, a Java-based digital image processing software developed by researchers at the Research Service Branch in Maryland, USA, and widely used in health and biology research.

### Statistical Analysis

One-way analysis of variance (ANOVA) and multiple comparison methods were performed. Datasets were expressed with 95% confidence intervals (CIs) and a significance level of *P* < 0.05.

### Ethical Considerations

This research was carried out following the approval by the Ethics Committee at the Faculty of Medicine, Padjadjaran University, Bandung (No.: 710/UN6.KEP/EC/2020). This study complied with the ARVO Statement for the Use of Animals in Ophthalmic and Vision Research and was approved by KEP Unpad (Padjadjaran University Research Ethics Committee.

##  RESULTS

Initially, 37 rats underwent habituation for 2 weeks before hyperglycemia was induced by intraperitoneal injection of 60 mg/kgBW STZ. Four rats were separated for the negative control group. The remaining 33 rats were divided into three groups and injected with STZ. After the injection, seven rats were excluded due to a minimal increase in blood glucose level. Six rats (four from group 3 [treated with metformin] and two from group 4 [treated with SGLT2 inhibitor] were found dead during the 8 weeks of drug administration. Enucleation was performed in 24 rats to obtain chorioretinal tissue samples (four rats from group 1, six rats from group 2, seven rats from group 3, and seven rats from group 4). The SGLT-2 and GLUT-1 expressions were examined using the Western blot technique.

### Blood Glucose Levels

The mean blood glucose levels of all groups, based on the treatment schedule, are shown in Figure 1.

### SGLT-2 Expression

**Figure 2:**
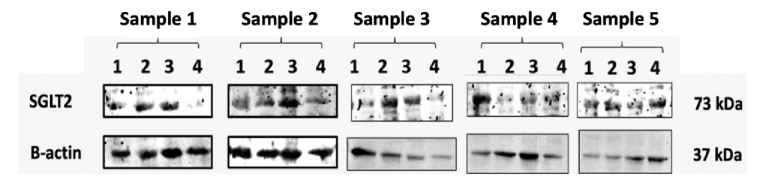
SGLT-2 expression based on Western blot examination. Each sample was taken from groups 1, 2, 3, and 4.

Examination was carried out on 20 mg of chorioretinal tissue obtained after enucleation. An internal control was used to minimize unavoidable variations in the experiment, such as inconsistent gel loading and transfer variations, thereby ensuring the reliability of data and results. To assess the expression of SGLT-2, β -actin was used as an internal control. Due to several obstacles during the blotting process, only 20 tape images, divided into four groups, were well-formed. This study found SGLT-2 expression in the chorioretinal tissue of rats, as shown in the Western blot in Figure 2.

**Figure 3:**
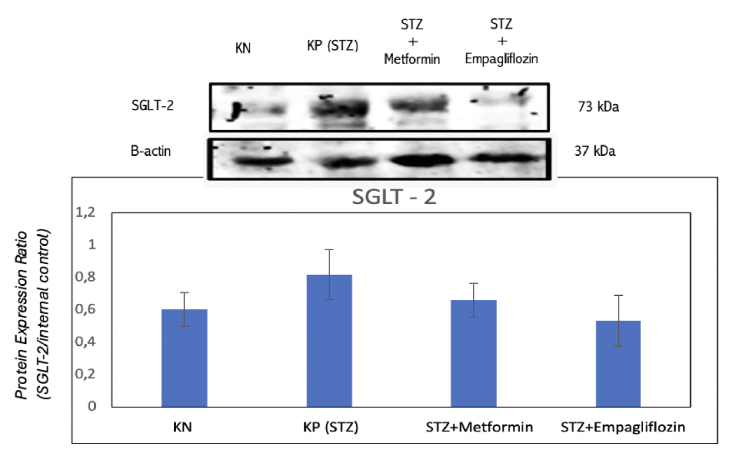
Average SGLT-2 expression in the four study groups. KN: nondiabetic rat group. KP (STZ): a group of diabetic rats without any treatment. STZ+METFORMIN: Metformin-treated diabetic rats. STZ+EMPAGLIFOZIN: Empagliflozin-treated diabetic rats.

Figure 3 shows that SGLT-2 expression is present in all rat groups, and administration of SGLT-2 inhibitors can suppress SGLT-2 expression in the chorioretinal tissue. The average expression of SGLT-2 can be seen in Figure 2. The average band thickness in this study indicated that SGLT-2 expression is higher in diabetic rats (positive control) compared to rats with normal glucose levels (negative control). Both metformin and empagliflozin have been shown to suppress SGLT-2 expression; however, the average reduction in SGLT-2 expression appears to be larger with empagliflozin than with metformin.

**Figure 4:**
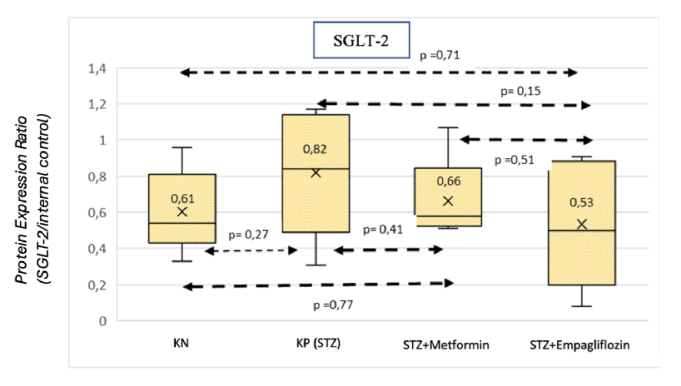
Results of multiple comparison analysis. KN: nondiabetic rat group. KP (STZ): a group of diabetic rats without any treatment. STZ+METFORMIN: Metformin-treated diabetic rats. STZ+EMPAGLIFOZIN: Empagliflozin-treated diabetic rats.

Multiple comparison analysis was carried out in each group [Figure 4]. The* P*-value from the multiple comparison analysis shows that the differences in each group are not statistically significant (*P* > 0.05).

### GLUT-1 Expression

**Figure 5:**
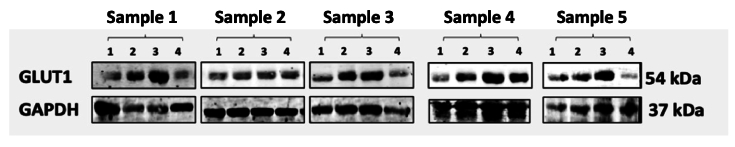
GLUT-1 expression band based on Western blot examination. Each sample was taken from groups 1, 2, 3, and 4.

**Figure 6:**
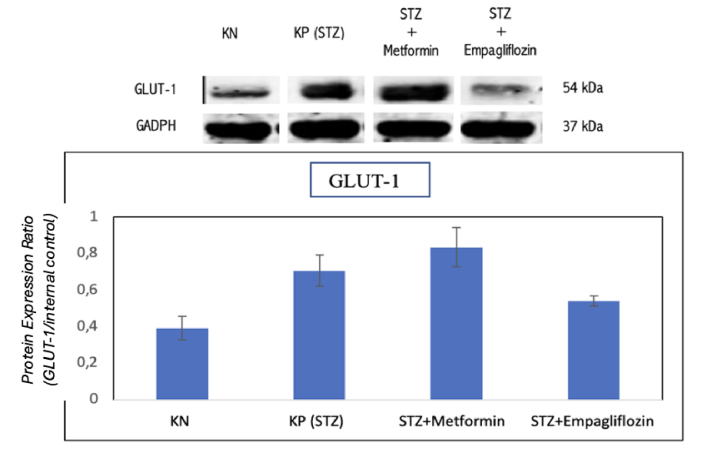
Average GLUT-1 expression across the four study groups. KN: nondiabetic rat group. KP (STZ): a group of diabetic rats without any treatment. STZ+METFORMIN: Metformin-treated diabetic rats. STZ+EMPAGLIFOZIN: Empagliflozin-treated diabetic rats.

GLUT-1 expression was found in all four study groups based on the examination of the chorioretinal tissue using the Western blot technique. The GLUT-1 expression band is presented in Figure 5. GLUT-1 expression did not change in the metformin group, but it decreased significantly in the empagliflozin group. Figure 6 illustrates the average GLUT-1 expression across the four study groups.

**Figure 7:**
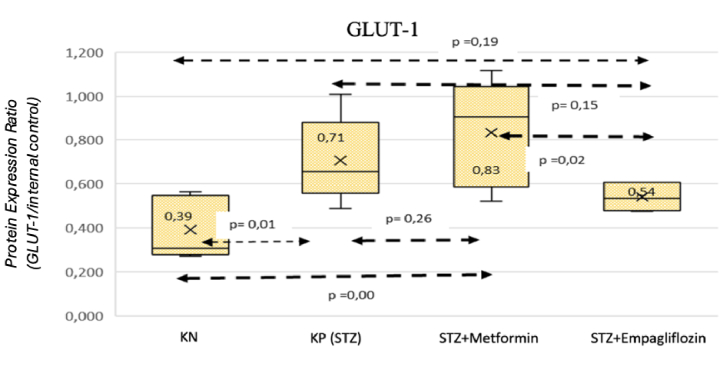
Results of multiple comparison analysis for GLUT-1 expression. KN: nondiabetic rat group. KP (STZ): a group of diabetic rats without any treatment. STZ+METFORMIN: Metformin-treated diabetic rats. STZ+EMPAGLIFOZIN: Empagliflozin-treated diabetic rats.

Multiple comparison analysis revealed a statistically significant difference in GLUT-1 expression between the positive control (STZ) and negative control groups (*P *= 0.01). In addition, a statistically significant difference was observed between the diabetic mouse groups treated with metformin and empagliflozin. Compared to rats treated with metformin (*P* < 0.05), empagliflozin-treated rats showed a lower level of GLUT-1 expression. The results of multiple comparison analysis are illustrated in Figure 7.

##  DISCUSSION

Among the many classes of antidiabetic drugs, SGLT-2 inhibitors possess a protective effect against retinal vascular damage, thus preventing the progression of DR and the occurrence of diabetic macular edema. Ott et al showed that the administration of SGLT-2 inhibitor dapagliflozin for 6 weeks provides beneficial effects on vascular remodeling.^[15]^ Takakura et al found no irregularities in the outer nuclear layer of the retina during treatment with SGLT2 inhibitor ipragliflozin.^[16]^ According to Dziuba et al, patients who had taken dapagliflozin for 20 years experienced a relative reduction (9.8%) in the DR incidence compared with standard care, whereas Tang et al did not find a higher risk of DR during the administration of SGLT-2 inhibitors compared with placebo.^[17,18]^

In this study, the positive control group, the metformin-treated group, and the empagliflozin-treated group experienced a decrease in blood glucose level, particularly after week 9. However, the reduction in the positive control group was only minor compared to the other groups. Additionally, the graph demonstrated that the decrease was even greater in diabetic rats treated with empagliflozin. Repeated-measures analysis confirmed that the difference between empagliflozin and metformin in reducing blood glucose levels was significant, with empagliflozin showing superior efficacy (*P* = 0.017). In a previous study, although SGLT-2 inhibitors were as effective as metformin, sulfonylureas, or sitagliptin in lowering blood glucose, they had a longer-lasting effect than the other antidiabetic drugs.^[19]^ However, further studies are required.

The expression of SGLT-2 in the renal cortex has been well-established in the literature.^[20]^ Wang et al found that the expression of mRNA and SGLT-2 protein was higher in the kidney biopsy of patients with diabetic nephropathy compared to diabetic mice.^[21]^ Although the same kind of expression seems to occur in the eye, there is still limited knowledge in this regard. Wakisaka et al reported the presence of SGLT-2 expression in cultured bovine retinal pericytes.^[22]^ Their findings correlate with the results of our research, as we found SGLT-2 expression in the chorioretina of both nondiabetic and diabetic rats, although not at a very high rate. Umino et al showed that SGLT-2 expression increased in cultured cells with high glucose levels due to the disruption of GLUT-2/importin 𖦹-1 and the increase in the Importin 𖦹-1/HNF-1𖦹 complex, which induced SGLT-2 transcription.^[23]^

The previous study by Wakisaka et al found that SGLT-2 expression was higher in diabetic rats compared to nondiabetic rats, and SGLT-2 inhibitors were able to suppress SGLT-2 expression in the eye. Compared to the other three groups, SGLT-2 expression was relatively low in the group of rats treated with empagliflozin, which may be due to the suppressive effect of empagliflozin on blood glucose levels. Empagliflozin may also have a direct effect on the retina, although the mechanism remains unknown. Further studies are required to support this hypothesis.

GLUT-1 is widely recognized as a glucose carrier that can cross the BRB, as it is mainly expressed in the vascular endothelial cells. GLUT-1 is responsible for glucose distribution in ganglion cells, photoreceptors, and Müller cells in the retina.^[24]^ In this study, GLUT-1 expression was found to be higher in the chorioretina of diabetic rats compared to nondiabetic rats.

GLUT-1 expression in the kidney has been studied extensively in the past. Evidence has shown that patients with type 1 DM and normal urine albumin levels had a decrease in GLUT-1 mRNA expression in the kidney glomerulus. In contrast, patients with type 1 DM and microalbuminuria exhibited an increase in GLUT-1 mRNA expression compared to nondiabetic patients.^[25]^ Additionally, studies on cultured cells suggest that GLUT-1 expression increases in podocytes and mesangial cells with higher glucose levels.^[26]^

On the other hand, the effect of diabetes on GLUT-1 expression in the retina remains largely unknown. Badr et al found a 50% reduction in GLUT-1 expression in the retina and blood vessels of STZ-induced diabetic rats.^[24]^ GLUT-1 expression was also higher in RPE cells compared to the neural retina. This finding does not align with our research, in that we observed a higher GLUT-1 expression in the chorioretinal layer of STZ-induced rats compared to non-diabetic rats.

Several theories have been proposed to explain the factors that influence GLUT-1 expression in the kidney. Heilig et al described that a reduction in nephron mass and the stretching of mesangial cells due to hypertensive glomeruli might increase GLUT-1 expression.^[26]^ Other contributing factors include the vasoactive effect of angiotensin II; activation of transforming growth factor beta (TGF-𖦹) and mitogen-activated protein kinase, which increase glucose influx by DAG–PKC pathway; increased vascular endothelial growth factor (VEGF) expression; and overexpression of platelet-derived growth factor (PDGF).^[26]^ In contrast, the change in GLUT-1 expression in diabetic retina has not been described yet. It is hypothesized that the glucose-lowering effect of empagliflozin and metformin may reduce GLUT-1 expression in the chorioretina, which is consistent with increased GLUT-1 expression in hyperglycemic environments. As a result, well-controlled blood glucose levels after SGLT-2 administration can normalize GLUT-1 expression in the chorioretina.

This study found that SGLT-2 expression decreased more upon empagliflozin administration compared to the other three groups, although the difference was not statistically significant. In addition, GLUT-1 expression also decreased after SGLT-2 inhibitor administration, suggesting a possible interaction between the actions of SGLT-2 and GLUT-1 in diabetic patients. Thus far, no study has explained the presence of this interaction. We hypothesize that this may be related to the decrease in blood glucose level after initiating treatment with SGLT-2 inhibitors. As shown in this study, SGLT-2 inhibitors are more effective in reducing blood glucose levels compared to metformin. As SGLT-2 and GLUT-1 expressions are higher in hyperglycemic tissue, SGLT-2 inhibitors may reduce GLUT-1 expression due to lower blood glucose post-therapy. However, further research is needed to elucidate possible interaction pathways between SGLT-2 and GLUT-1.

To date, this study is the first to compare the effects of two antidiabetic drugs on SGLT-2 and GLUT-1 expressions in the chorioretina. Compared to SGLT-2 in renal physiology, research on SGLT-2 in the eye is still limited. Nevertheless, this study may shed light on the interplay between SGLT-2 expression and DR, as well as provide valuable insights into the potential use of SGLT-2 inhibitors to prevent DR.

This study has several limitations, such as a short observation period and the use of oral medication with a calculated equivalent dose. In addition, we examined the chorioretina, instead of specifically in retinal blood vessels, the RPE, or the neuronal layer of the retina.

##  Financial Support and Sponsorship

None.

##  Conflicts of Interest

None.
